# Application of CRISPR/Cas9 Technology in Rice Germplasm Innovation and Genetic Improvement

**DOI:** 10.3390/genes15111492

**Published:** 2024-11-20

**Authors:** Jijin Chen, Zhening Miao, Deyan Kong, Anning Zhang, Feiming Wang, Guolan Liu, Xinqiao Yu, Lijun Luo, Yi Liu

**Affiliations:** Shanghai Agrobiological Gene Center, Shanghai 201106, China; cjj@sagc.org.cn (J.C.); mzn@sagc.org.cn (Z.M.);

**Keywords:** gene editing, CRISPR/Cas9, rice, genetic improvement

## Abstract

Improving the efficiency of germplasm innovation has always been the aim of rice breeders. Traditional hybrid breeding methods for variety selection rarely meet the practical needs of rice production. The emergence of genome-editing technologies, such as CRISPR/Cas9, provides a new approach to the genetic improvement of crops such as rice. The number of published scientific papers related to “gene editing” and “CRISPR/Cas9” retrievable on websites both from China and other countries exhibited an increasing trend, year by year, from 2014 to 2023. Research related to gene editing in rice accounts for 33.4% and 12.3% of all the literature on gene editing published in China and other countries, respectively, much higher than that on maize and wheat. This article reviews recent research on CRISPR/Cas9 gene-editing technology in rice, especially germplasm innovation and genetic improvement of commercially promoted varieties with improved traits such as disease, insect, and herbicide resistance, salt tolerance, quality, nutrition, and safety. The aim is to provide a reference for the precise and efficient development of new rice cultivars that meet market demand.

## 1. Introduction

Rice (*Oryza sativa* L.) is one of the world’s major food crops and a staple food for over 50% of the global population. It is widely planted in various regions and holds significant social and economic importance. With rapid population growth, the demand for food crops has greatly increased. According to the Food and Agriculture Organization (FAO) of the United Nations, the global demand for agricultural products is expected to grow by about 70% by 2050. Therefore, the annual grain production needs to increase from the current 2.1 billion tons to approximately 3 billion tons to feed the predicted world population [1]. The biotic stresses caused by field diseases and pests affect crop yields to varying degrees. Diseases such as rice blast, bacterial leaf blight, and sheath blight significantly limit rice yield. The brown planthopper is a huge challenge in rice production worldwide, causing serious yield losses in rice. Extreme climatic conditions such as high temperatures, droughts, and floods occur frequently worldwide, and these abiotic stresses reduce crop yields by nearly 50%, further threatening human food security [2]. Therefore, cultivating high-yielding, high-quality, and environmentally adaptable crop varieties has currently become the goal pursued by breeders, and improving plants’ tolerance to biotic and abiotic stresses is one of the focuses of agricultural research [3]. However, traditional crop breeding methods require processes such as crossing, backcrossing, and selection, which involve long breeding cycles, complex steps, and low efficiency, severely limiting the speed of breeding. Random mutagenesis, naturally occurring mutations, and classical breeding techniques require prolonged periods and sometimes do not generate individuals with the desired phenotype. Gene editing is a simple and efficient technique for inserting, deleting, or replacing gene bases at the genomic level. Previous studies have shown that gene editing can be used for crop trait improvement while reducing the effects of abiotic stresses [4,5]. The emergence and development of gene-editing technologies such as CRISPR/Cas9 have provided new approaches for efficient germplasm innovation and genetic improvement in crops such as rice.

## 2. The Emergence and Development of CRISPR/Cas9

### 2.1. The Emergence of the CRISPR/Cas System

In 1987, Ishino et al. discovered regular repeats of DNA sequences in *Escherichia coli* [6]. Mojica also discovered similar repetitive sequences in 2000 when analyzing archaeal DNA sequences [7]. In 2005, Mojica discovered that prokaryotes contain short repetitive DNA sequences called Clustered Regularly Interspaced Short Palindromic Repeats (CRISPRs), which form the prokaryotic adaptive immune system together with CRISPR-associated (Cas) genes. This system can recognize and perform targeted cleavage on the specific sequences of bacteriophages that have previously invaded the bacteria [8]. Another study further demonstrated that CRISPR/Cas is part of the adaptive defense system of bacteria and archaea against invasion by phages and exogenous plasmid DNA and that it includes CRISPR/Cas9-related Cas enzymes [9]. Based on phylogenetics, comparative genomics, and structural analysis, CRISPR/Cas systems can be divided into three types: type I systems containing the Cas3 protein, type II systems containing the Cas9 protein, and type III systems containing the Cas10 protein [10,11].

CRISPR/Cas is an important genome-editing tool developed based on the CRISPR system and widely used in various organisms, such as bacteria, yeast, and plants. CRISPR/Cas9 consists of two basic components: gRNA (guide RNA), which identifies the target gene sequences, and Cas9 (CRISPR-associated protein 9), which is an endonuclease which can create double-stranded DNA breaks in the genome. The double-strand breaks (DSBs) of chromosomes can be repaired through two DNA repair mechanisms, i.e., homology-directed repair (HDR) and non-homologous end-joining (NHEJ) [12]. The DSBs generated by Cas9 induce NHEJ repair, which produces small random insertions or deletions at the cleavage site [13]. These DSBs can also be repaired in a targeted manner through HDR, producing precise genomic modifications at the cleavage sites [14].

### 2.2. The Development of the CRISPR/Cas System

The CRISPR technology was first applied to mammalian cells in 2013, with the successful achievement of Cas9-induced DSBs in both mouse and human cells [15]. Due to its simple design, low vector construction costs, and high editing efficiency, the CRISPR/Cas9 gene-editing technology has been widely used to study gene functions in animals, plants, and microorganisms, making it the mainstream gene-editing technology over a short period of time [16]. The functions of the CRISPR/Cas9 components have also been explored and optimized, leading to the development of base-editors capable of performing single-base editing and guided-editing techniques capable of conducting precise genome modifications [17,18]. Different types of genome-editing tools can mediate DNA DSBs, triggering HDR and NHEJ repair mechanisms to achieve base deletion or insertion of the target gene. Compared to techniques such as ZFNs (zinc-finger guided-editing techniques) and TALENs (transcription activator-like effector nucleases), CRISPR/Cas9 exhibits higher gene editing efficiency and accuracy, reducing off-target effects while enhancing editing efficiency [19].

The advancement of the CRISPR/cas9 technology has made gene editing a versatile tool capable of knocking-in or making structural variations (SVs) to enhance the function of genes or reduce the expression of a gene [20,21]. The emergence and development of gene-editing technology have also enhanced the efficiency of breeding strategies such as de novo domestication, non-fusion reproduction, and polyploid induction, providing multiple technical options for the breeding strategies of crops such as rice [22,23].

### 2.3. Trends in the Publishing of CRISPR/Cas-Related Papers

**Figure 1:**
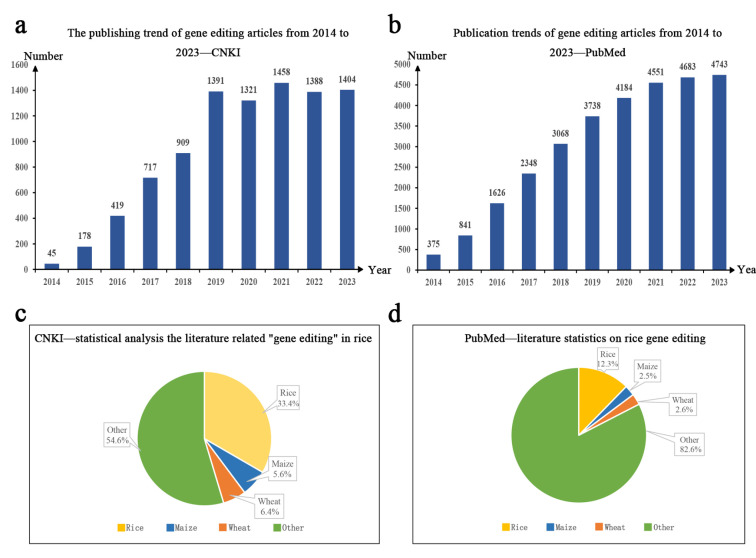
Publication trend of gene editing-related studies between 2014 and 2023. (**a**,**b**) show the number of articles published every year related to gene-editing technology on websites CNKI and PubMed during the 10-year period from 2014 to 2023; (**c**,**d**) show the proportion of three major staple crops, namely rice, maize, and wheat, in gene editing-related articles during the 10-year period. The (**c**) graph contains the data and analysis derived from CNKI, and the (**d**) graph represents the data and analysis derived from Pubmed. Yellow represents rice, blue represents maize, orange represents wheat, and green represents other crops; all raw data for the analytical process can be found in the Supplementary Material from Tables S1–S4.

Scientific and technological journals are important sources for knowledge transmission and academic communication [24], and the quantitative analysis of journal literature can effectively reflect the development trend of a certain research field. China National Knowledge Infrastructure (CNKI) and PubMed are well-known literature review platforms, with massive amounts of data available for reference and analysis. This article aimed to understand the publication status of gene editing papers in China and internationally and accurately determine the development trends and progress in gene-editing technology. Using “gene editing” as the search term, 9230 and 30,157 papers related to gene editing, published between 2014 and 2023, were retrieved from CNKI and PubMed, respectively, with an increasing trend in publication in subsequent years (Figure 1a,b). To further analyze the research progress in staple crop gene editing, we used “gene editing” and “CRISPR/Cas9” as the search terms and “crops” as the discipline category to download and summarize the literature published between 2014 and 2023, obtaining a total of 2163 publications on CNKI. Using text analysis methods, the “titles” of 2163 papers were extracted for further analysis. After comparison and deduplication, 1631 valid papers were obtained. Among them, 544, accounting for 33.4%, were related to rice gene editing, while 105, accounting for 6.4%, were related to maize (*Zea mays* L.) gene editing, and 91, accounting for 5.6%, were related to wheat (*Triticum aestivum* L.) gene editing (Figure 1c).

Similarly, by summarizing and analyzing research related to “gene editing” and “plant CRISPR/Cas9” on the English literature indexing website PubMed, 4995 valid studies were obtained, with studies on rice, maize, and wheat accounting for 12.3%, 2.5%, and 2.6% of the total, respectively (Figure 1d). According to the results obtained from the two databases, gene editing studies on rice involved both basic and applied research, as opposed to those featuring maize and wheat, indicating that rice gene editing plays a dominant role as the basis for gene editing application to crops. Rice is a major cereal crop and an important model crop species. The CRISPR/Cas9 technology not only promotes gene functional studies in rice but also provides an efficient approach for variety improvement.

## 3. Application of CRISPR/Cas9 in Rice Genetic Improvement

### 3.1. Biotic Stress Resistance

#### 3.1.1. Rice Blast Resistance

Rice blast is among the major diseases that threaten rice growth, reducing the total global rice yield by between 10% and 30% annually [25]. At present, chemical control is the main approach used to prevent and control rice blast; however, large-scale chemical usage causes land pollution and pesticide resistance in the pathogen. In recent years, the CRISPR/Cas9 gene-editing technology has been effectively employed to improve the disease resistance of rice by editing disease-resistant genes to generate new disease-resistant varieties. Wang et al. conducted the functional knockout of the *Pi21* gene in the Nanjing 9108 rice variety using the CRISPR/Cas9 technology to obtain mutants, laying the foundation for developing new rice lines with broad-spectrum resistance to rice blast [26]. Similarly, Zhou et al. created new hybrid rice germplasm with broad-spectrum resistance to rice blast by knocking out the blast disease susceptibility genes *Bsr-d1*, *Pi21*, and *ERF922* in the sterile line Longke 638S [27]. Wu and Peng et al. used gene-editing technology to knock out the *pid3* and *pi21* genes of the rice variety Dalixiang, creating high-quality materials resistant to rice blast disease without genetic modification or changes in rice quality [28,29].

#### 3.1.2. Bacterial Blight Resistance

Bacterial blight is a vascular disease caused by the Gram-negative bacterium *Xanthomonas oryzae* pv. Oryzae (Xoo). It can reduce rice yield by between 20% and 30%, or up to 50% in severe cases, and even result in no grain harvest [30]. Zhu et al. used a single Cas9/gRNA to target the homologous sequences of the *Xa13* and *Xa25* genes, disrupting the function of the target genes and inducing bacterial blight resistance in five rice varieties, including Yuxiang Youzhan, indica restorer line Shuhui 143, and indica sterile line Zhinong S. This provided a promising germplasm resource for developing rice varieties with lasting resistance to bacterial blight [31]. Zeng et al. used CRISPR/Cas9 to modify the corresponding coding region of *OsSWEET14* in the rice variety Zhonghua 11 and generated nine different *OsSWEET14* mutant alleles. The mutants exhibited not only broad-spectrum resistance to Asian bacterial strains but also strong resistance to African bacterial strain *AXO1947* [32].

#### 3.1.3. Brown Planthopper Resistance

The frequent occurrence of pests in rice fields poses a huge danger for the world’s food security. Currently, the biological methods for agricultural pest control mainly involve mining and utilizing insect-resistant genes, as well as breeding rice varieties with strong disease resistance. The brown planthopper is a typical monophagous herbivore that feeds on the phloem and poses a significant threat to rice production and global food security. Lu et al. conducted an integrated transcriptome and metabolome analysis and found that *OsSPL10* knockout could enhance direct and indirect resistance to *BPH*, and this could serve as a potential target for rice genetic improvement for resistance to brown planthoppers [33]. Tang et al. used the rice line Xiushui 11 to construct a homozygous *OsLRR-RLK18* knockout line and found that the egg production of brown planthoppers on this line was significantly reduced compared to un-edited Xiushui 11, indicating that *OsLRR-RLK18* plays an essential role in regulating rice resistance to brown planthoppers [34]. Lu et al. knocked out the *CYP71A1* gene in the Xidao 1 rice variety, generating a mutant with significant insect resistance [35].

### 3.2. Tolerance to Abiotic Stresses

#### 3.2.1. Salt Tolerance

Salt stress is one of the unfavorable factors caused by global climate change that affects the growth of crops. Rice is very sensitive to salt stress, especially during the seedling stage. Identifying salt-tolerance genes and developing genetically modified germplasm for rice breeding are effective ways to reduce the impact of salt stress on rice production. Zhang et al. obtained two T2 homozygous mutant lines with significantly enhanced salt tolerance using Cas9-OsRR22-gRNA, thereby improving the salt tolerance of the japonica rice variety WPB106 [36]. Sheng et al. improved the salt tolerance of next-generation hybrid rice lines by combining the CRISPR/Cas9-mediated targeted editing of *OsRR22* with hybrid vigor utilization in varieties 733-3B and Huazhan [37]. Furthermore, Hang et al. used the CRISPR/Cas9 technology to create a new *OsRR22* mutant germplasm with improved salt tolerance from the three-line restorer line R192, providing a reference for improving salt tolerance in rice [38]. Kumar et al. used CRISPR/Cas9 for the targeted editing of the DST gene of the indica rice variety MTU1010 and found that the mutant had a higher tolerance to salt stress, with increased yield [39].

#### 3.2.2. Herbicide Resistance

Weeds are an important factor affecting rice production since they compete with rice for resources such as nutrients, water, and sunlight. In addition, the presence of weeds also increases the occurrence of pests and diseases and can reduce rice yield by more than 40% [40]. The appropriate use of herbicides can reduce the impact of weeds on rice yield to a certain extent. At present, most herbicides that can kill weeds also cause certain damages to rice plants. Using the CRISPR/Cas9 technology to quickly and accurately edit relevant genes regulated by herbicides and cultivating herbicide-resistant rice varieties are the most promising means to control weeds. Jiang et al. used optimized base-editing techniques ePE3max and ePE5max to edit the *EPSPS* gene in the japonica rice variety, generating homozygous mutations (T173I and A174V) and a heterozygous mutation P177S (TAP-IVS), thus laying a solid foundation for the non-transgenic breeding of glyphosate resistance in rice [41]. Li et al. used the CRISPR/Cas9-mediated NHEJ pathway to insert endogenous *EPSPS* genes in rice to obtain rice materials containing glyphosate-resistant genes [42]. Furthermore, Liu et al. generated two new single-mutation sites, I1879V and W2125S, in the coding region of the herbicide-resistant gene *OsACC* through CRISPR-mediated base screening of rice callus, generating the same aryloxyphenoxypropionic acid herbicide resistance previously reported in the C2186R site [43,44]. Sun et al. used a CRISPR/Cas9 plasmid vector with two gRNAs to modify the *ALS* sequence in the rice genome through the directed insertion and substitution of bases and obtained herbicide-resistant rice varieties containing a discontinuous point mutation in *ALS* [45]. Kuang et al. used base-editing-mediated gene evolution (BEMGE) to artificially change the *OsALS1* gene in rice cells, thereby generating two sites with varying degrees of tolerance to the herbicide sodium dipyridamole. The study also edited the P171F site of the *OsALS* gene in Nanjing 46 using base-editing to obtain mutant materials resistant to bispyribac-sodium, demonstrating the enormous potential of the BEMGE method in creating important genetic variations in target genes for crop improvement [46]. Wu et al. used CRISPR/Cas9 and CRISPR/Cas12a to edit the 3′UTR region of the *OsHPPD* gene and created a rice line resistant to the HPPD herbicide in the background of the Yangjing 3012 variety [47]. Using an optimized adenine base editor (ABE), TadA9, Yan et al. simultaneously edited four endogenous herbicide-resistant genes in the commercial rice variety Nanjing 46 and generated SNPs for multiple herbicide-resistant genes, providing a potential approach for controlling weeds in rice fields [48].

### 3.3. Improving Rice Quality

#### 3.3.1. Grain Shape

Currently, cultivating rice with suitable grain shapes and low chalkiness is important for improving the appearance quality of rice. Rice with slender grains has a lower chalkiness and a better appearance quality but lower whole-grain rate and grain weight. Zhao et al. used gene-editing technology to knock out the *GS9* gene in the Yandao 8 rice variety. The *gs9* mutant had increased grain length, a significantly reduced chalkiness rate and degree, and a significantly improved appearance quality [49]. Usman et al. used the CRISPR/Cas9 technology to edit the *GS3* gene in the japonica rice variety TP309 and obtained stable long-grain rice mutants [50].

#### 3.3.2. Chalkiness Degree and Chalky Grain Rate

Liu et al. simultaneously edited *Chalk5*, *Gn1a*, *GW2*, and *TGW3* genes in the high-quality variety Nanjing 9108 using the CRISPR/Cas9 technology, obtaining different allelic variation combinations which significantly reduced chalkiness and improved grain weight and yield [51]. Tan et al. used the CRISPR/Cas9 technology to edit the region near the ACII motif in the promoter region of the gene encoding the slender grain type SLG7/GL7/GW7 and created three new SLG7 allelic variations that significantly reduced chalkiness in the background of Wuyunjing 30. Another study indicated that the AH2 transcription factor could bind the SLG7 promoter and inhibit its expression. Knocking out the ACII element or adjacent fragments of the SLG7 promoter can also weaken the binding ability of AH2 to the SLG7 promoter, thereby enhancing the expression level of SLG7, increasing the length–width ratio of the grain and reducing rice chalkiness [52]. GWD1/LSE1 encodes a glucan-hydrating kinase that regulates starch metabolism in rice leaves. The CRISPR/Cas9 technology was used to edit the promoter region of GWD1, creating two down-regulated mutants, gwd1-1 and gwd1-2. The chalky rate and chalkiness degree of these two lines significantly decreased, with an improved appearance quality and no significant changes in other traits such as plant type and grain weight [53,54].

#### 3.3.3. Rice Fragrance

Fragrance is one of the important indicators that determine the edibility of rice grain. 2-acetyl-1-pyrroline (2-AP) is the main source of rice fragrance, and its biosynthesis is mainly controlled by the recessive gene *OsBADH2,* encoding betaine aldehyde dehydrogenase. Qi et al. edited exons 2 and 7 of the *OsBADH2* gene of non-aromatic japonica rice varieties Jia 58 and Xiushui 134 using the CRISPR/Cas9 technology and generated multiple mutants. The 2-AP content was significantly increased in four T2-edited materials in the background of Xiushui 134 [55]. Zhu et al. performed targeted aroma improvement for Ningjing 50 using CRISPR/Cas9. The grain of the mutant had increased aroma, with no significant changes in the chalky rate and chalkiness degree [56].

#### 3.3.4. Low Content of Amylose Starch

The taste quality directly reflects consumers’ acceptance of rice and is an important indicator for evaluating rice quality. The taste quality of rice is determined by various characteristics, from its production to processing. Rice with good taste quality is characterized by a good appearance, round grains, a strong aroma, and moderate softness and hardness. Zeng et al. created new allelic variations conferring varying degrees of reduced amylose content, such as *Wx^a-dA^*, *Wx^a-dC1^*, *Wx^a-dC2^*, and *Wx^a-dC3^*, by editing the promoter region of the *Wx* gene in indica rice variety TFB [57]. The *Du13* gene can regulate the splicing efficiency of rice endosperm gene *Wx^b^* by activating the C2H2 zinc-finger protein, thereby affecting the amylose content in rice. Cai et al. used gene-editing technology to perform a knockout experiment on the japonica rice variety Koshihikari and indica rice variety Nanjing 11 to create a mutant, *Y1*, with a decreased amylose content and an increased taste value [58]. Transcription factors can bind to the promoter sequence of target genes to regulate their gene expression. Many reports have shown that the amylose content in rice can be regulated by editing transcription factors to regulate the expression of the *Wx* gene [59,60,61].

### 3.4. Nutrition and Safety of Rice

#### 3.4.1. Low Glutelin Content

The endosperm is the main edible component of rice, composed of starch (80~90%), protein (7~10%), and lipids (1%). The content and composition of each component in the endosperm directly affect the edibility and taste quality of rice [62]. Genetic improvement of the regulatory genes of each component in the endosperm can improve the edibility and taste quality of rice. Yang et al. used the CRISPR/Cas9 technology to simultaneously knock out eight highly expressed genes from four subgroups (GluA, GluB, GluC, and GluD) of rice glutenin genes. The mutant rice showed varying degrees of reduced protein content [63]. Chen et al. designed three sgRNAs to target nine glutenin genes and created nine homozygous lines without T-DNA fragments in the background of Wuyunjing 7, among which most lines showed varying degrees of reduced glutenin content [64]. Wang et al. used gene-editing technology to edit two amino acid transporter genes, *OsAAP6* and *OsAAP10*, in multiple lines, such as Yangjing 158 and Nanjing 9108, significantly reducing the glutenin content [65].

#### 3.4.2. Reducing Heavy Metal Accumulation in Rice

Heavy metals, even in trace amounts, pose a huge threat to human health. Plants can absorb heavy metals, and the long-term consumption of foods with excessive heavy metal contents can increase the risk of diseases [66]. Excessive levels of heavy metals in rice, such as rice with excessive cadmium, not only affect its quality but also seriously impact human health. Cultivating low-cadmium rice varieties and reducing cadmium content in rice have become new breeding goals for rice breeders. Studies have shown that the loss of function of the cadmium uptake and transport gene OsNramp5 can effectively reduce the accumulation of cadmium in rice. Hu et al. used the CRISPR/Cas9 technology to knock out the cadmium uptake and transport gene OsNramp5 in Chuanhui 491 material and created a new rice germplasm with low cadmium accumulation, providing new genetic resources for accelerating the cultivation of suitable rice varieties which can be planted in cadmium-contaminated areas [67]. Similarly, Yang et al. knocked out the OsNRAMP5 gene in two japonica rice varieties, Nanjing 46 and Huaidao 5, using CRISPR/Cas9, and obtained three OsNRAMP5 mutants without transgenic markers. Compared to wild-type plants, the accumulation of cadmium and manganese was significantly reduced in the roots, shoots, and grains of the mutants [68]. Chang et al. knocked out the OsNRAMP2 gene in Zhonghua 11, significantly reducing the distribution of cadmium in rice leaves, straw, and grains [69]. Xu et al. used the CRISPR/Cas9 technology to edit the promoter region of OsLsi1 and the C-terminal coding sequences of OsLsi1/OsLsi2 in the Zhongjiazao 17 and Jiahe 212 varieties, thereby providing a method of reducing arsenic accumulation in rice grains without affecting grain yield [70].

## 4. Future Prospects

After more than a decade of continuous development and innovation in gene-editing technologies, particularly CRISPR/Cas9, the rapid, targeted improvement of traits such as stress resistance, quality, nutrition, and safety has been achieved in various rice varieties. The technology greatly improved the efficiency of germplasm innovation and became an important genetic improvement method for rice. In addition, cutting-edge biological breeding technologies such as de novo domestication, non-fusion reproduction, and haploid induction, developed through gene-editing technology, also provide unlimited possibilities for the molecular improvement of crops. However, some challenges still limit the application of gene editing, including editing efficiency, intellectual property rights of gene-edited germplasms, and transgenic safety of the process of germplasm innovation:

(1) The CRISPR/Cas9 technology is currently the most widely used method to edit target genes and improve crops by knocking out functional genes that negatively regulate target traits. However, the number of such major functional genes with negative regulatory effects is limited, and most traits are regulated synergistically by multiple genes. Therefore, there is a need for further functional genomic studies of rice to explore gene resources with important breeding value and iteratively upgrade the CRISPR system to enhance the ability and efficiency of site-directed mutagenesis, large-fragment knock-in, and multi-gene editing.
(2) Protecting intellectual property protects breeding innovation. Currently, commercially promoted rice varieties are mainly developed through traditional hybrid breeding with balanced, comprehensive traits. Gene-edited germplasms based on these varieties are more easily accepted on the market and can achieve industrialization, which also better reflects the applicability of gene-editing technology. However, the edited germplasm and original basic varieties belong to substantial derived varieties. A number of countries, including the United States and the United Kingdom, have enacted stringent legislation and regulations to safeguard the intellectual property rights of seeds. These jurisdictions also extend the same protective measures to the seeds of gene-edited crops, while progressively relaxing controls on gene-edited crops. The revised version of the 2022 Seed Law in China specifically emphasizes the establishment of a substantial derived variety system, with the aim of acknowledging and encouraging original innovation and establishing a reasonable system for distributing benefits.
(3) Self-segregation can eliminate exogenous fragments in gene-edited progeny materials, which also ensures gene editing without introducing exogenous genes. Genome-editing technology is not accepted or is still under debate in some countries because the resulting genotypes are considered “GMOs” and are not accepted commercially. Some countries, such as the United States, Japan, and Australia, have adopted relatively flexible regulatory policies for gene-edited crops [71]. The Chinese Ministry of Agriculture and Rural Affairs issued the Guidelines for the Safety Evaluation of Agricultural Gene-Edited Plants (Trial) and the Detailed Rules for the Evaluation of Agricultural Gene-Edited Plants in 2022 and 2023, respectively. In 2023, Shandong BellaGen Biotechnology Co., Ltd., obtained China’s first safety certificate of agricultural gene editing for high-oleic soybeans generated through gene editing, further accelerating the industrial application of gene-editing technology in crops.

The advent of gene editing has ushered in a new era of biological breeding technology, paving the way for a revolutionary shift in rice variety selection and breeding. This revolutionary approach, dubbed “Breeding 4.0”, promises to usher in a new era of precision agriculture, enabling the design and breeding of crops with unparalleled precision and efficiency. This technology has the potential to transform the global food supply and modernize the seed industry, ensuring a sustainable and reliable food source for the future.

## Data Availability

All the data generated or analyzed during this study are included in this published article.

## Supplementary Materials

The following supporting information can be downloaded at https://www.mdpi.com/article/10.3390/genes15111492/s1: Table S1: CNKI_Title_Doi; Table S2: PubMed_Title_Doi; Table S3: CNKI_Analysis_Result; Table S4: Pubmed_Analysis_Result.
